# Scientific authorship by gender: trends before and during a global pandemic

**DOI:** 10.1057/s41599-022-01365-4

**Published:** 2022-10-04

**Authors:** Ji-Young Son, Michelle L. Bell

**Affiliations:** School of the Environment, Yale University, New Haven, CT USA

**Keywords:** Education, Science, technology and society

## Abstract

Many fields of science are still dominated by men. COVID-19 has dramatically changed the nature of work, including for scientists, such as lack of access to key resources and transition to online teaching. Further, scientists face the pandemic-related stressors common to other professions (e.g., childcare, eldercare). As many of these activities fall more heavily on women, the pandemic may have exacerbated gender disparities in science. We analyzed self-identified gender of corresponding author for 119,592 manuscripts from 151 countries submitted January 2019 to July 2021 to the Institute of Physics (IOP) portfolio of 57 academic journals, with disciplines of astronomy and astrophysics, bioscience, environmental science, materials, mathematics, physics, and interdisciplinary research. We consider differences by country, journal, and pre-pandemic versus pandemic periods. Gender was self-identified by corresponding author for 82.9% of manuscripts (*N* = 99,114 for subset of submissions with gender). Of these manuscripts, authors were 82.1% male, 17.8% female, and 0.08% non-binary. Most authors were male for all countries (country-specific values: range 0.0–100.0%, median 86.1%) and every journal (journal-specific values range 63.7–91.5%, median 83.7%). The contribution of female authors was slightly higher in the pandemic (18.7%) compared to pre-pandemic (16.5%). However, prior to the pandemic, the percent of submissions from women had been increasing, and this value slowed during the pandemic. Contrary to our hypothesis, we did not find that manuscript submissions from women decreased during the pandemic, although the rate of increased submissions evident prior to the pandemic slowed. In both pre-pandemic and pandemic periods, authorship was overwhelmingly male for all journals, countries, and fields. Further research is needed on impacts of the pandemic on other measures of scientific productivity (e.g., accepted manuscripts, teaching), scientific position (e.g., junior vs. senior scholars), as well as the underlying gender imbalance that persisted before and during the pandemic.

## Introduction

The COVID-19 pandemic has dramatically changed the way people live and the nature of work, including for the scientific community. Many governments worldwide implemented mitigation efforts such as social distancing, lockdown, and stay-at-home measures to reduce COVID-19 transmission. These mitigation measures have affected many aspects of our lives, with the scientific community shifting to working from home, work disruption through loss of laboratory facilities and other resources, and transition to online teaching. As a result of mitigation measures (e.g., closure of schools and daycare centers), many scientists had to work at home along with increased domestic responsibilities, childcare, and eldercare. Further, students experienced additional stress and anxiety due to the pandemic, which may have disproportionately affected women as female faculty spend more time performing service to the university compared to men (Suitor et al., 2001; Guarino and Borden, 2017). A survey of scientists in six languages found that work hours for teaching and administration increased during the pandemic more for women than men, and that female scholars were more concerned than men about potential negative consequences of the COVID-19 pandemic on long-term career progress and publication record (Heo et al., 2022). Surveys of medical faculty in the United States found that men were twice as likely as women to have accelerated productivity during the pandemic (Ellinas et al., 2022) and women had increased teaching load (Lufler and McNulty, 2022).

Researchers in Africa reported spending more time working during the pandemic than earlier, with male researchers having more time than women for publications, patents, and consulting (Adekola et al., 2022). However, studies were not consistent with a survey of Canadian academic researchers reporting overall fewer hours worked during the pandemic, particularly for parents of young children (Gordon and Presseau, 2022).

Gender disparities in academia involve gender imbalance at work and home regarding burden and responsibility of domestic labor within households (Suitor et al., 2001; Schiebinger and Gilmartin, 2010; Jolly et al., 2014; Johnson et al., 2020). Female scientists spend more hours on household duties such as food preparation, childcare, domestic labor, and other household responsibilities than male scientists (Schiebinger and Gilmartin, 2010; Jolly et al., 2014).

Gender disparities in authorship of scientific works existed prior to the pandemic, particularly for some disciplines (e.g., surgery, computer science, physics, mathematics), certain positions (e.g., senior authors), the most prestigious journals, single author manuscripts, and invited articles (West et al., 2013; Holman et al., 2018; Hornstein et al., 2022). Differences in scientific productivity by gender can be impacted by career length and leaving the field (Huang et al., 2020). Women are more likely to experience disagreements regarding scientific authorship and to have their contributions devalued by both men and women (Ni et al., 2021).

We hypothesized that the pandemic has likely amplified these existing disparities, and that female scientists are more likely to be impacted by the pandemic in relation to scientific authorship due to increased family responsibilities. Many studies have examined how gender disparities in authorship of scientific papers were affected by the pandemic. Overall, the findings of these studies were mixed, with many findings indicating higher scientific productivity by men compared to women during the pandemic compared to pre-pandemic periods, but inconsistent results across studies (Table 1). These differences in results may relate to methodologies, disciplines, and the categorization of scientific productivity (e.g., article submission vs. published article).

**Table 1:** Key studies on scientific authorship by gender in relation to the COVID-19 pandemic.

Article	Journals/disciplines (no. of Journals or Publication Databases)	No. of manuscripts^a^	Method to assess authors’ gender^b^	Key results on COVID-19’s influence on authorship by gender
*This study*	*Astronomy/astrophysics, biosciences, environmental science, interdisciplinary, materials, mathematics, physics (57 journals)*	*119,592*	*Self-identified*	*Women contributed a higher % of articles during the pandemic than pre-pandemic, however this value had been increasing over time prior to the pandemic*
Abramo et al. (2022)	General (3 databases)	153,231	Algorithm	Larger decrease in articles for men than women for corresponding author, with variation by region
Anabaraonye et al. (2022)	Radiation oncology (1 journal)	458	Perceived gender through internet search	No statistically significant decrease in the overall proportion of women publishing
Anderson et al. (2020)	Medicine (1 database)	15,843 COVID articles, 316,367 “control” articles	Algorithm	COVID-19 papers have lower % female first authors than papers pre-pandemic Inconclusive results for last author and overall authorship of COVID-19 papers by gender
Ayyala and Trout (2022)	Pediatric radiology (1 journal)	1108	Authors’ knowledge, name, internet search	No significant difference in female authorship over time for first, last or corresponding authors
Babicz et al. (2021)	Clinical neuropsychology (4 journals, additional analysis of 40 articles from 9 journals)	1,018. Additional analysis of 40 articles	First name and US and UK Census data, website	% of women lead/corresponding authors did not change comparing the pandemic and pre-pandemic
Bell and Fong (2021)	Public health (1 journal)	1767	Algorithm	Submission rates increased more for men than women during pandemic compared to pre-pandemic period for the US
Biondi et al. (2021)	Agricultural economics (4 journals)	5366	Algorithm	Submissions increased equi-proportionately by gender No evidence of near-term disruption in publications
Bittante et al. (2022)	COVID-19 (1 database)	1448	Website photos	Men were more represented than women as first and last authors
Chen and Seto (2021)	Urban land science (1 database)	1582	Algorithm, pronouns, photos	Productivity increased for women and decreased for men during pandemic compared to pre-pandemic period
Cook and Gupta (2021)	Obstetrics and gynecology (6 journals)	655	Authors assessment of name and websites	No difference in gender of first author during pandemic compared to pre-pandemic period
Cui et al. (2022)	Social science (1 database)	41,858	Algorithm, authors’ assessment of professional webpages	Preprints by women increased during pandemic compared to pre-pandemic period, but declined in relation to men’s productivity
Cushman (2020)	Thrombosis, hemostasis, and vascular biology (1 journal)	178	Self-report	No difference in authorship by gender for first and corresponding authors
DeFilippis et al. (2021)	Cardiology (4 journals)	7627	Algorithm, pronouns	Proportion of women first and senior authors higher in 2020 than 2019
Dolan and Lawless (2020)	Political science (1 journal)	108 during pandemic	Unspecified	Higher % of female authors during the pandemic than pre-pandemic
Fox and Meyer (2020)	Ecology (6 journals)	6042	Algorithm	No evidence of disproportionate impact on female authors due to pandemic
Gayet-Ageron et al. (2021)	Biomedicine COVID-19 (11 journals)	63,259	Algorithm	Women less likely to be first author on COVID-19 papers compared to pre-pandemic papers Gender distributions of authorship were similar for non-COVID-19 manuscripts for pandemic and pre-pandemic periods
Gershengorn et al. (2022)	Pulmonary and critical care (4 journals)	8332	Algorithm	No change in proportion of female first or senior authors during pandemic compared to pre-pandemic period Articles with female senior author less likely to be accepted for non-COVID papers
Harris et al. (2022)	School psychology (3 journals)	804	Algorithm, authors’ assessment	No significant difference in gender of first author comparing pandemic and pre-pandemic period
Ipe et al (2021)	Transfusion medicine (4 journals)	1024	Algorithm, manual verification through unspecified means	Lower % of female first authors in the pandemic period No statistical change for senior authors
Jemielniak et al. (2022)	21 disciplines (2813 journals)	266,409	Algorithm	No significant differences between men and women publication patterns, although patterns differed by discipline
Jordan and Carlezon (2021)	Neuropsychopharmacology (1 journal)	1940	Pronouns, website, photographs, first name	% of women similar during the pandemic and pre-pandemic periods for corresponding author
Liu et al. (2022)	COVID-19 (1 database)	332,458	Algorithm	Gender disparities in authorship increased in pandemic compared to pre-pandemic period, then returned to pre-pandemic levels Papers from teams with females in a leading role were less cited in both periods, but this disparity increased during pandemic
Lerchenmüller et al. (2021)	Life sciences (3426 journals)	42,898 COVID articles, 483,232 “control” articles	Databases (e.g., first name and Social Security Administration data)	Gender disparity in authorship widened under pandemic Patterns differed by country
Madsen et al. (2022)	Medicine, biology, chemistry, clinical medicine (1 database)	2,113,108	Algorithm	Gender difference in publication productivity increased from 2019 to 2020 Widening gender gap for early career and mid-career scientists Most prominent gender gap for highly productive authors and those in biology or clinical medicine
Mah et al. (2022)	Gynecologic oncology (2 journals)	3022	Pronouns, Google image results and websites for first names, algorithm	Men were more represented as authors in all time periods No immediate impact of the early pandemic on the gender distribution of authors
Marescotti et al. (2022)	Neuroscience, neurology, psychiatry (1 journal)	796	Algorithm	% of authors who were women decreased during pandemic No differences in trends of gender disparities between first, middle, or last authors
Mogensen et al. (2021)	Radiology (1 journal)	752	Authors’ knowledge, internet search	Proportion of female first authors during pandemic lower than during pre-pandemic period, although difference not statistically different Similar results for corresponding author
Muric et al. (2021)	Biomedicine (62 journals)	78,980	Algorithm	Proportion of female authors declined overall for first author and last author Gender disparities differed by country
Nguyen et al. (2021)	Ophthalmology (65 journals)	119,457	Algorithm	COVID-19 articles had lower % women authors (first, middle, last) than predicted
Orchard et al. (2022)	Occupational and environmental health (1 journal)	3531	Algorithm	Increased productivity during pandemic compared to pre-pandemic period for men, but less so for women
Quak et al. (2021)	Medical imaging (50 journals)	7073	Algorithm	% of authors who were women slightly higher during pandemic than pre-pandemic period Female authors for COVID-19 papers were overrepresented at the lowest ranked journal
Ribarovska et al. (2021)	Brain behavior and immunity (1 journal)	Not specified	First name, pronouns, appearance	Female authorship slightly lower during the pandemic compared to the pre-pandemic period
Ryskina et al. (2022)	Medicine (7 journals)	2856	Pronouns, first name and US Social Security databases	No differences in proportion of articles by gender of lead author for pandemic and pre-pandemic periods, although baseline disparities remained
Squazzoni et al. (2021)	Health and medicine, life sciences, physical sciences and engineering, social sciences and economics (2329 journals)	1,983,799	Algorithm	Women submitted proportionally fewer manuscripts during pandemic
Ucar et al. (2022)	General (5 databases)	307,902	Algorithm	Proportion of male authors for preprints increased during pandemicHigher proportion of male authors in COVID-19 preprints
Williams et al. (2021)	Pediatric medicine (1 journal)	1,521	Algorithm	Proportion of women authors decreased during pandemic compared to pre-pandemic period Gender disparities differed by country
Wooden and Hanson (2022)	Earth and space science (23 journals)		American Geophysical Union (AGU) member profiles, algorithm	No difference in proportion of male and female corresponding authors comparing pandemic and pre-pandemic periods
Wright et al. (2022)	Family medicine (1 journal)	4325	Algorithm	Submissions increased more for men than women

^a^The number of articles may be preprints, submissions, accepted manuscripts, and/or published manuscripts depending on the study design. The number used in analysis may be lower (e.g., inability to assign gender). Some works also investigated reviewer invitations and editorial boards.

^b^Algorithms are based on variables such as author’s first name and country of residence.

In the 38 previous studies described in Table 1, a variety of approaches were used to identify the gender of authors: algorithms, authors’ assessment of website photos, authors’ assessment of first names, pronouns, databases (e.g., gender distribution of first name in Social Security Administration data), and authors’ knowledge of the papers’ authors. Most studies used an algorithm, often based on first name and country of the author’s organization. Such algorithms are useful but have limitations such as inaccurate predictions for Chinese names (Sebo, 2022), difficulty with unusual names and nongendered names, and linkage to country of the author’s organization rather than their country of birth or citizenship. Only two of the 38 previous articles used self-identified gender in any capacity; an evaluation of 23 earth and space science journals considered self-identified gender for members of the American Geophysical Union for 51% of authors, used an algorithm for 30%, and did not predict gender for 19% (Wooden and Hanson, 2022), and a small study of one journal and <200 articles used self-reported gender (Cushman, 2020).

Here we investigated gender differences regarding the authorship of scientific manuscripts under the COVID-19 pandemic. We hypothesized that the distribution of authorship by gender changed over time in relation to the pandemic, with higher productivity for men, given that many of the impacts on work described above (e.g., childcare) may be more likely to fall on women than men. To establish baseline conditions, we assessed patterns of authorship by gender prior to the pandemic and compared those to patterns after the pandemic began. We examined whether changes in patterns of scientific authorship by gender under the pandemic differ by region or discipline. We used submissions to scientific journals of Institute of Physics (IOP) Publishing as an indicator of scientific work conducted, although we recognize that submission of manuscripts is not the only nature of scientific work (e.g., teaching, reviewing articles, advising, public outreach and community engagement). Our work differs from earlier efforts in that, to the best of our knowledge, this is the first large such study for which gender was self-identified by the authors. Also, our work differs from some earlier efforts given the broad scope of journals included in the IOP portfolio.

## Methods

We obtained data on manuscript submissions to the IOP for Jan. 2019 to July 2021, through a Confidential Information Agreement. IOP journals focus on physics, materials sciences, biosciences, astronomy and astrophysics, environmental sciences, mathematics, and education, as well as interdisciplinary research (IOP, 2020). Many of the journals are published in coordination with professional societies and research organizations. The IOP manuscript submission process requests gender of the corresponding author, with options of male, female, or non-binary. Authors also have the option to not disclose gender. The dataset included, for each article submitted, the name of the journal, the self-identified gender of the corresponding author, and the date (month, year) submitted. In the initial dataset, 119,592 manuscript submissions were included.

The journal was specified for all submissions. The data includes submissions to 57 journals. We assigned journals to disciplines based on IOP categorization and journal descriptions: astronomy and astrophysics (2 journals), bioscience (15 journals), environmental science (9 journals), interdisciplinary (7 journals), materials (22 journals), mathematics (7 journals), and physics (29 journals). Some journals contributed to more than one category.

Analysis by country excluded 77 submissions that did not have country specified for the organization of the corresponding author. In total 152 countries were included in the initial dataset (*N* = 119,515 submissions with country specified). Regional analysis was based on United Nations regions: Africa with five subregions, Asia with five subregions, Europe with four subregions, Latin America and the Caribbean with four subregions, Oceania, and Northern America.

While there exists no universal definition for the start of the COVID-19 pandemic, we used an approach that accounts for different patterns of the pandemic by country. For consistency in analysis across countries, the start date of the pandemic was defined separately for each country as the date with 50 or more confirmed cases (University of Oxford, 2020). We assigned midday of the month as the date of article’s submission day as our data includes information on the submission year and month, but not the day. A difference-in-difference approach was used to compare monthly submissions by men and women during the pandemic to that of the pre-pandemic period. We conducted analyses using R Statistical Software 4.1.0 (R Foundation for Statistical Computing, Vienna, Austria) and Excel (Microsoft, Redmond, WA).

## Results

Our initial database had 119,592 manuscript submissions. Of these, gender of corresponding author was self-identified as male for 81,381 submissions (68.0%), female for 17,655 (14.8%), non-binary for 78 (0.1%), and unknown [prefer not to say or no response] for 20,478 (17.1%). Of manuscripts for which gender was identified (*N* = 99,114), corresponding authors were 82.1% male, 17.8% female, and 0.08% non-binary. The frequency of undisclosed gender differed across journals (average 14.9% of submissions, median 15.1%), with the lowest at 1.9% for *IOP SciNotes* and the highest at 32.9% for *Materials Research Express*. Across the six global regions, the rate of nondisclosure of gender ranged from 13.4% (Oceania) to 17.8% (Northern America).

### Gender of authorship in relation to the pandemic

**Fig. 1:**
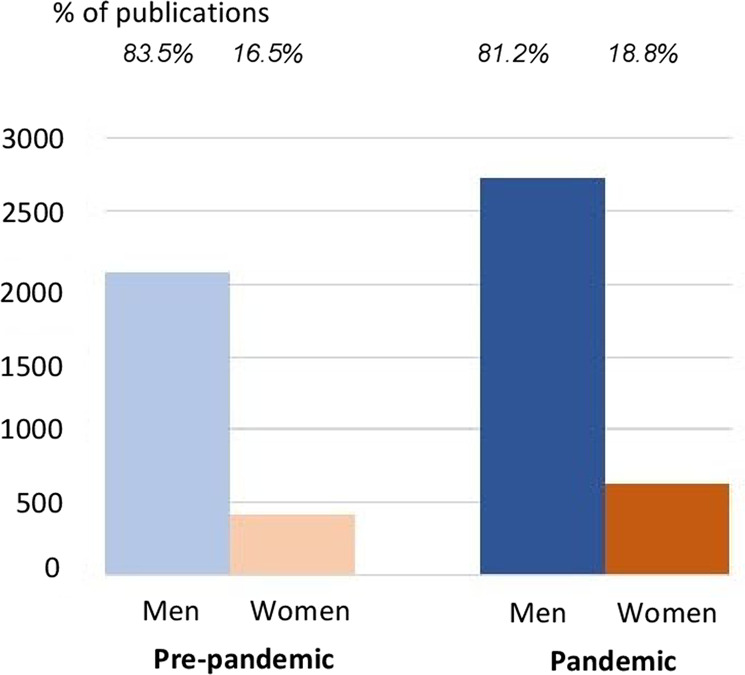
Average number of submissions/month by gender and pandemic period (comparing men and women). The percent of corresponding authors who self-identified as non-binary was 1.6% in the pre-pandemic period and 3.2% in the pandemic period.

Men comprised a larger percentage of corresponding authors than women for the pre-pandemic period and pandemic period (Fig. 1). Overall, women contributed 16.5% of articles before the pandemic and 18.8% of articles during the pandemic. These values should be interpreted with caution given the uncertainty in the start of a pandemic and the variability in submission rates. Both men and women submitted *more* manuscripts per month during the pandemic compared to the pre-pandemic period. However, prior to the pandemic, submission rates for both men and women were increasing, and during the pandemic this rate of increase slowed for both men and women. Before the pandemic, women and men were averaging approximately 30 and 91 more submissions each month compared to the month before, respectively. Still, the change in submission rates over time suggests that the timing of the pandemic coincided with a lowering of scientific productivity overall, in the sense that previous trends of increased submissions did not persist, even though the number of articles/month was higher during the pandemic. In other words, while the percent of corresponding authors that were women increased during the pandemic compared to the pre-pandemic period, this value was increasing *faster* before the pandemic compared to during the pandemic. The influence of the pandemic on authorship was statistically different for men and women based on difference-in-difference estimates with fixed estimates for time, accounting for country or journal.

### Gender of authorship by region and country

Supplemental Table S1 shows the patterns of authorship by gender and world region. Among the six main regions, considering the submissions with gender specified, the lowest female representation for was Latin America and the Caribbean at 16.0% and highest for Africa at 19.2%. Non-binary authorship comprised a very small percent of authors at 0.0 to 0.1% across the six regions. By subregion, within Africa female authorship was lowest for Eastern Africa (3.5%), within Asia was lowest for Central Asia (8.2%), and within Europe was lowest for Eastern Europe (16.9%). Males comprised the majority of authors for every region and every subregion.

Analysis by country for those with at least 30 submissions is shown in Supplemental Table S2. Men had more submissions than women for all 87 countries. The largest female representation was for Ghana and Tunisia (38.1 and 35.51% of the submissions with gender specified, respectively) and lowest for Democratic People’s Republic of Korea (i.e., North Korea) and the State of Palestine (0% women).

The percent of corresponding authors who were male decreased from 83.5% pre-pandemic to 81.2% during the pandemic, considering submissions for which gender was specified, indicating that women had an increase in submissions compared to men. Table 2 shows the percentages of men and women in submissions/month comparing pre-pandemic and pandemic periods by region. While overall the percent of women authors increased by 2.2%, this value remained low in the pandemic period (increase from 16.5 to 18.7%), and men had more submissions than women for all regions in both time periods. The largest difference was for Europe, for which the percent of corresponding authors who were women increased by 2.5%, which was also the region with the highest contributions from women in the pandemic period. Women had larger increases in submission rates than did men for all regions except Africa, for which this value decreased 0.7%. Difference-in-difference analysis did not identify a significant shift in the gender of authors in relation to the pandemic by region or country.

**Table 2:** Percent of corresponding authorship by gender for pre-pandemic and pandemic periods, by world region.

	Pre-pandemic period			Pandemic period			Change
World region	% Male	% Female	% Non-binary	% Male	% Female	% Non-binary	% Female
Africa	80.2	19.7	0.1	80.9	19.1	0.0	−0.7
Asia	84.1	15.9	0.0	81.6	18.3	0.1	2.4
Europe	82.4	17.5	0.1	79.9	20.0	0.2	2.5
Latin America and the Caribbean	84.5	15.5	0.0	83.5	16.4	0.1	0.8
Oceania	79.5	17.3	3.2	78.7	18.9	2.5	1.5
Northern America	82.2	17.7	0.1	80.2	19.7	0.1	2.0
Overall	83.4	16.5	0.1	81.1	18.7	0.1	2.2

The percent change for men is −1 times that of women (e.g., −2.4% change for Asia for men). Percentages are based on submissions with gender specified. Submissions without country specified, from countries with unspecified pandemic start date, or without specified gender were excluded. *N* = 97,957.

### Gender authorship by discipline

Supplemental Fig. S1 and Supplemental Table S3 show the percent of authors by gender by journal category, for the seven disciplines in the IOP portfolio. Considering the submissions with gender specified, the highest male authorship was for astronomy and astrophysics at 89.6% and the lowest for bioscience journals at 79.01%. Further, every individual journal had primarily male authorship (range 63.7 to 91.5%) (Supplemental Table S4).

Table 3 shows the percent of corresponding authors who were men or women during the pre-pandemic and pandemic periods by discipline. The percent of authors who were women increased for all journal categories with the exception of astronomy and astrophysics for which there was no change. Interdisciplinary journals had the largest increase with women contributing 10.1% of articles prior to the pandemic and 15.2% during the pandemic. Difference-in-difference analysis did not identify a significant shift in the gender of authors by discipline.

**Table 3:** Percent of corresponding authorship by gender for pre-pandemic and pandemic periods, by journal category.

	Pre-pandemic period			Pandemic period			Change
Category (no. of journals)	% Male	% Female	% Non-binary	% Male	% Female	% Non-binary	% Female
Astronomy and astrophysics (2)	89.5	10.3	0.2	89.6	10.3	0.1	0.0
Bioscience (15)	80.6	19.3	0.0	78.0	22.0	0.1	2.6
Environmental science (9)	80.5	19.5	0.1	78.3	21.5	0.1	2.1
Interdisciplinary (7)	89.8	10.1	0.1	84.7	15.2	0.1	5.1
Materials (22)	82.7	17.2	0.0	80.0	19.9	0.1	2.7
Mathematics (7)	88.4	11.5	0.1	86.6	13.2	0.2	1.7
Physics (29)	85.1	14.8	0.1	83.6	16.3	0.1	1.5

The percent change for men is −1 times that of women (e.g., −2.6% change for bioscience for men). Some journals contributed to more than one category. Submissions from countries with unclear pandemic period information or submissions without specified gender were excluded.

## Discussion

This study found gender imbalances in scientific manuscript submissions before the pandemic, which persisted during the pandemic. Males comprised the vast majority of corresponding authors for all regions, countries, journals, and disciplines. Overall, submission rates during the pandemic were higher compared to pre-pandemic period for both men and women. Contrary to our initial hypothesis, submission rates from women increased at a higher rate than those of men for all regions other than Africa and all disciplines other than bioscience. Despite larger percent increase in submissions for women than men, during the pandemic period women still comprised only 18.7% of corresponding authors compared to 81.1% of men. However, the historical trends of increased authorship from women slowed under the pandemic, indicating the pandemic did influence women differently from men and suggest that without the pandemic, the disparity in authorship would be less.

We had hypothesized that the distribution of authorship by gender changed over time in relation to the pandemic, with higher productivity for men, given that many impacts of the pandemic on work such as childcare may be more likely to fall on women than men. However, our results found that the productivity of women scientists, based on submission of manuscripts, actually increased more than that of men. Even with these increases, the pre-existing gender imbalances in productivity persisted for all journals, disciplines, countries, and regions.

Previous studies on how the pandemic affects scientific authorship by gender are not consistent, with some studies finding no effect and others suggesting that the pandemic exacerbated pre-existing gender imbalances in scientific productivity. Most previous studies reported that women had lower submissions than men during the pandemic (Table 1). For example, a study found that the proportion of women authors during the pandemic dropped significantly for biomedical research (Muric et al., 2021). A study using data on all Elsevier journals found that women submitted fewer manuscripts than men during the pandemic (Squazzoni et al., 2021). They suggested that productivity by women was impacted more substantially by the pandemic than that of men due to additional demands for time and effort (e.g., family duties). However, some studies reported findings more similar to our study (Cushman, 2020; Dolan and Lawless, 2020). An evaluation of the influence of the pandemic on manuscript submissions and editor and reviewer performance at ecology journals did not find disproportionate impacts of the pandemic on women authors and reviewers (Fox and Meyer, 2020). A study of articles in urban land science found that during the pandemic, submissions increased 51% for women and declined 11% for men (Chen and Seto, 2021). We found that women constituted a higher percentage of corresponding authors during the pandemic than previously for all disciplines other than astronomy and astrophysics, which also had the lowest contributions from women. However, our findings also suggest that that the percentage of women authors would have been even higher without the pandemic, as the trend of increasing percent of submissions by women slowed during the pandemic. Further, under both the pandemic and pre-pandemic periods, men had a higher percentage of corresponding authors for all disciplines and journals.

We found that the women contributed fewer manuscripts than men for all world regions for both the pandemic and pre-pandemic periods. Women had a higher percentage of articles during the pandemic compared to the pre-pandemic for all regions except Africa. Our findings differed from those of some previous studies. One study examined the change in gender gap in academic authorship globally, finding a significant reduction in women’s first authorships in almost all geographic areas, except some Asian countries with no change (China) or small increase (Taiwan, South Korea) (Lerchenmüller et al., 2021). Another study also observed a significant decline in the number of papers published by women as first authors and found that this increased gender gap was persistent across the 10 countries with the highest number of researchers (Muric et al., 2021).

Differences between our study results on gender gap for scientific authorship in relation to the pandemic and those of some earlier works may relate to several methodological differences. First, many other studies examined a single journal and we considered a larger number of publications with 57 journals, although several earlier studies also examined multiple journals (Table 1). Second, we examined corresponding author, whereas some other work considered first author or all authors. Thus, our findings may be more applicable for senior scholars, although corresponding authors are not necessarily the most senior authors. Third, our study and others focused on specific disciplines, such as ecology or urban land science. We analyzed data for manuscripts in astronomy and astrophysics, biosciences, environmental science, materials, mathematics, physics, and interdisciplinary studies. The gender gap in science overall is decreasing, but male authors still constitute a higher fraction of authors of scientific papers for some disciplines such as the physical sciences, mathematics, and engineering (Sanderson, 2021). Thus, the focus of the journals analyzed here may represent more male-dominated areas of science. Fourth, we focused on submitted manuscripts rather than published articles. Fifth, the time periods used to define pre-pandemic and pandemic periods differed by study. Finally, gender in our data was self-identified by the authors, but was estimated by algorithms or other means for most earlier works.

Strengths of this work include a large number of manuscripts (over 100,000) for 57 journals and self-identification of gender. To the best of our knowledge, this is the first large study of scientific authorship by gender in relation to the pandemic using data on self-identified gender. Earlier works primarily used an algorithm, such as those based on first name and country of their organization, or other methods such as assessment of photographs, pronouns, and authors’ knowledge of the individuals. All these methods are less reliable than self-identified gender. While algorithms to predict gender are useful, they are limited for unusual names, authors whose country of residence is different from their original country, and nongendered names. A study of Chinese first names found that gender prediction algorithms had errors of 43 to 90% (Sebo, 2022), whereas a similar study based on names from Switzerland found much better performance with errors at 1.8 to 17.7% (Sebo, 2021).

However, our study has some limitations. We did not have information on the scholar’s age, position, or research experience, and these may relate to differences in how the pandemic affected scientific productivity due to factors such as higher childcare responsibilities for some age groups of scholars than others, with differences also relating to the age of the child (Krukowski et al., 2021). A study investigating differences in academic productivity by gender and child age during the pandemic reported that faculty with younger children (0–5 years) had significantly fewer work hours compared to all other groups of scholars including those with no children or those with older children. Also, they found that faculty with children age 6 years or older or without children reported significant increases or stable academic productivity compared to scholars with younger children. Some studies reported a sudden drop in the proportion of female authors in the early stages of pandemic and then a subsequent gradual increase, which may partially be explained by adjustment to the new environment (Muric et al., 2021).

Data on self-identified gender was not available for 17.8% of participants. The analyses of submissions with gender specified assumes that the distribution of gender within those who chose not to disclose gender was similar in the pre-pandemic and pandemic periods. Likelihood to disclose gender may in fact differ by gender. Due to sample size, we were able to compare men and women, but not non-binary authors who represented 0.1% of submissions. Due to stigma and discrimination, lesbian, gay, bisexual, transgender, queer, and other (LGBTQ + ) individuals may be less likely to disclose their gender, and barriers to disclosure have been identified in other settings (e.g., health care, friends and family, school, workplace) (Ragins et al., 2007; Mansh et al., 2015; Brooks et al., 2018; Schrimshaw et al., 2018; Lyons et al., 2021). Further, we recognize that there are genders other than the male, female, and non-binary options provided to authors for selection and that gender identity is a continuum (Castleberry, 2019; Gulgoz et al., 2022).

Our information on submission date included month and year, not specific date, due to data availability. Although we considered country-specific information on the pandemic, there is no precise start date of the pandemic, and there can exist substantial heterogeneity regarding pandemic mitigation measures and resources within and across countries. In addition to differences in local and government policies, authors’ organizations can have widely different responses to the pandemic in terms of shifts to online teaching, remote work, support for mental health and wellbeing, support for childcare, access to laboratories and other resources) (Babbar and Gupta, 2021). Our findings on patterns of authorship may not be generalizable to other types of authorship (e.g., first author, all authors, age, position). While we analyzed 57 journals from a range of disciplines, many scientific disciplines are not reflected here (e.g., humanities) and there may be differences within subfields. We analyzed manuscript submissions, rather than accepted publications. Thus, while our analysis reflects work conducted, it does not address differences in which articles are accepted. This study only considers one aspect of scientific productivity, which includes more than manuscripts, such developing methodologies, writing grant proposals, reviewing manuscripts, training and mentoring students and junior colleagues, and engaging in public outreach; these other facets of productivity may have gender inequities that differ from those of manuscript submissions. These inequities may have been affected by the pandemic in different ways than manuscript submission.

## Conclusion

Our study analyzing data on manuscript submissions to the IOP found that women’s submissions as corresponding author were higher during the pandemic compared to the pre-pandemic period, in contrast with trends in men’s productivity, although pre-pandemic trends of increasing authorship by women slowed during the pandemic, which suggests that without the pandemic the contribution from women may have been higher. Gender imbalances in scientific productivity existed both before and during the pandemic, as corresponding authors for manuscript submissions were overwhelming male across all regions, countries, disciplines, and journals, however, we found a complex relationship in which women’s submissions increased, but were still likely influenced by the COVID-19 pandemic, thereby exacerbating the disparities by gender in productivity for this facet of scholarship. Further research considering various contributing factors that may affect this relationship (e.g., childcare resources, type of authorship) is needed, including work on differences for accepted manuscripts and other forms of scientific work such as mentorship and teaching.

## Supplementary information

Supplemental Material

## Data Availability

Data were obtained through a Confidential Information Agreement with IOP and are thereby not publicly available. The tables presented here and in the Supplemental Material provide detailed data summaries.
