# Supplementary material for: Scientific authorship by gender: trends before and during a global pandemic
Source: Humanit Soc Sci Commun. 2022 Oct 4;9(1):348. doi: 10.1057/s41599-022-01365-4 (PMC9529602; doi:10.1057/s41599-022-01365-4)
Supplement: Supplementary file 1 — Supplemental Material [file 41599_2022_1365_MOESM1_ESM.docx]

**Scientific authorship by gender: trends before and during a global pandemic**

Ji-Young Son and Michelle L. Bell

Supplemental Table S1. Corresponding authorship by gender and world region

Supplemental Table S2. Corresponding authorship by gender and country

Supplemental Table S3. Corresponding authorship by gender and journal category

Supplemental Table S4. Corresponding authorship by gender and journal

Figure S1. Boxplot of percent of submissions with male corresponding author, by journal category

Supplemental Table S1. Corresponding authorship by gender and world region

|  | Considering all submissions | | | | | Considering submissions with gender specified | | | |
| --- | --- | --- | --- | --- | --- | --- | --- | --- | --- |
| World Region (# countries) | No. submissions | % Male | % Female | % Non-binary | Not specified | No. submissions | % Male | % Female | % Non-binary |
| Africa (35) | 4685 | 68.1 | 16.2 | 0.0 | 15.6 | 3952 | 80.7 | 19.2 | 0.1 |
| Eastern Africa (12) | 341 | 89.4 | 3.2 | 0.0 | 7.3 | 316 | 96.5 | 3.5 | 0.0 |
| Middle Africa (3) | 218 | 80.3 | 10.1 | 0.0 | 9.6 | 197 | 88.8 | 11.2 | 0.0 |
| Northern Africa (6) | 3192 | 64.3 | 20.1 | 0.0 | 15.7 | 2692 | 76.2 | 23.8 | 0.0 |
| Southern Africa (5) | 461 | 65.5 | 11.7 | 0.2 | 22.6 | 357 | 84.6 | 15.1 | 0.3 |
| Western Africa (9) | 473 | 75.5 | 7.0 | 0.0 | 17.5 | 390 | 91.5 | 8.5 | 0.0 |
| Asia (45) | 75,583 | 68.3 | 14.3 | 0.0 | 17.4 | 62420 | 82.7 | 17.3 | 0.1 |
| Central Asia (4) | 165 | 81.8 | 7.3 | 0.0 | 10.9 | 147 | 91.8 | 8.2 | 0.0 |
| Eastern Asia (8) | 43,483 | 67.1 | 14.5 | 0.1 | 18.3 | 35519 | 82.2 | 17.8 | 0.1 |
| Southeastern Asia (9) | 2626 | 67.3 | 16.6 | 0.0 | 16.1 | 2202 | 80.2 | 19.8 | 0.0 |
| Southern Asia (6) | 24,078 | 70.2 | 13.2 | 0.0 | 16.6 | 20078 | 84.2 | 15.8 | 0.0 |
| Western Asia (18) | 5231 | 69.2 | 16.2 | 0.0 | 14.5 | 4474 | 81.0 | 19.0 | 0.0 |
| Europe (42) | 22,343 | 67.3 | 15.9 | 0.1 | 16.7 | 18619 | 80.8 | 19.0 | 0.2 |
| Eastern Europe (10) | 4997 | 69.5 | 14.2 | 0.2 | 16.1 | 4191 | 82.8 | 16.9 | 0.2 |
| Northern Europe (11) | 5286 | 66.9 | 15.6 | 0.1 | 17.4 | 4364 | 81.0 | 18.9 | 0.1 |
| Southern Europe (13) | 4476 | 67.2 | 17.5 | 0.1 | 15.2 | 3797 | 79.3 | 20.6 | 0.1 |
| Western Europe (8) | 7584 | 66.4 | 16.2 | 0.1 | 17.4 | 6267 | 80.3 | 19.6 | 0.1 |
| Latin America and the Caribbean (21) | 4259 | 70.7 | 13.5 | 0.1 | 15.7 | 3590 | 83.9 | 16.0 | 0.1 |
| Caribbean (4) | 43 | 53.5 | 23.3 | 0.0 | 23.3 | 33 | 69.7 | 30.3 | 0.0 |
| Central America (5) | 1132 | 72.2 | 12.3 | 0.1 | 15.5 | 957 | 85.4 | 14.5 | 0.1 |
| Sandwich Islands (2) | 77 | 79.2 | 9.1 | 0.0 | 11.7 | 68 | 89.7 | 10.3 | 0.0 |
| South America (10) | 3007 | 70.2 | 13.9 | 0.1 | 15.8 | 2532 | 83.4 | 16.5 | 0.1 |
| Oceania (6) | 1549 | 70.4 | 16.2 | 0.0 | 13.4 | 1341 | 81.3 | 18.7 | 0.0 |
| Northern America (2) | 11,096 | 66.5 | 15.6 | 0.1 | 17.8 | 9119 | 80.9 | 18.9 | 0.1 |
| No country specified | 77 | 76.6 | 18.2 | 0.0 | 5.2 | 73 | 80.8 | 19.2 | 0.0 |

*Note*: Gender refers to self-identified gender of the corresponding author. Gender not specified refers to authors who selected “prefer not to say” or did not answer the question on gender. N=119,592 submissions.

Supplemental Table S2. Corresponding authorship by gender and country

|  | Considering all submissions | | | | | Considering submissions with gender specified | | | |
| --- | --- | --- | --- | --- | --- | --- | --- | --- | --- |
| Country | No. submissions | % Male | % Female | % Non-binary | Not specified | No. submissions | % Male | % Female | % Non-binary |
| Algeria | 628 | 59.6 | 19.7 | 0.2 | 20.5 | 499 | 74.9 | 24.8 | 0.2 |
| Argentina | 338 | 64.2 | 19.5 | 0.0 | 16.3 | 283 | 76.7 | 23.3 | 0.0 |
| Armenia | 54 | 87.0 | 5.6 | 0.0 | 7.4 | 50 | 94.0 | 6.0 | 0.0 |
| Australia | 1371 | 70.1 | 16.3 | 0.0 | 13.6 | 1184 | 81.2 | 18.8 | 0.0 |
| Austria | 421 | 70.5 | 15.4 | 0.0 | 14.0 | 362 | 82.0 | 18.0 | 0.0 |
| Azerbaijan | 31 | 83.9 | 16.1 | 0.0 | 0.0 | 31 | 83.9 | 16.1 | 0.0 |
| Bangladesh | 455 | 85.1 | 4.0 | 0.0 | 11.0 | 405 | 95.6 | 4.4 | 0.0 |
| Belarus | 51 | 68.6 | 11.8 | 0.0 | 19.6 | 41 | 85.4 | 14.6 | 0.0 |
| Belgium | 466 | 73.6 | 13.5 | 0.0 | 12.9 | 406 | 84.5 | 15.5 | 0.0 |
| Brazil | 2046 | 70.0 | 14.3 | 0.1 | 15.5 | 1728 | 82.9 | 17.0 | 0.1 |
| Bulgaria | 73 | 68.5 | 9.6 | 0.0 | 21.9 | 57 | 87.7 | 12.3 | 0.0 |
| Cameroon | 206 | 80.1 | 10.7 | 0.0 | 9.2 | 187 | 88.2 | 11.8 | 0.0 |
| Canada | 1467 | 68.9 | 15.7 | 0.1 | 15.3 | 1242 | 81.4 | 18.5 | 0.1 |
| Chile | 315 | 78.7 | 7.0 | 0.0 | 14.3 | 270 | 91.9 | 8.1 | 0.0 |
| China | 36509 | 64.9 | 15.9 | 0.1 | 19.1 | 29522 | 80.2 | 19.7 | 0.1 |
| Colombia | 221 | 73.8 | 10.4 | 0.0 | 15.8 | 186 | 87.6 | 12.4 | 0.0 |
| Croatia | 74 | 59.5 | 20.3 | 0.0 | 20.3 | 59 | 74.6 | 25.4 | 0.0 |
| Cyprus | 54 | 68.5 | 13.0 | 0.0 | 18.5 | 44 | 84.1 | 15.9 | 0.0 |
| Czech Republic | 491 | 72.3 | 13.0 | 0.0 | 14.7 | 419 | 84.7 | 15.3 | 0.0 |
| Denmark | 261 | 71.6 | 13.4 | 0.0 | 14.9 | 222 | 84.2 | 15.8 | 0.0 |
| Egypt | 1642 | 67.1 | 18.7 | 0.0 | 14.2 | 1409 | 78.2 | 21.8 | 0.0 |
| Estonia | 58 | 77.6 | 5.2 | 0.0 | 17.2 | 48 | 93.8 | 6.3 | 0.0 |
| Ethiopia | 251 | 93.2 | 1.2 | 0.0 | 5.6 | 237 | 98.7 | 1.3 | 0.0 |
| Finland | 368 | 64.7 | 17.7 | 0.0 | 17.7 | 303 | 78.5 | 21.5 | 0.0 |
| France | 1937 | 64.0 | 17.2 | 0.1 | 18.7 | 1574 | 78.7 | 21.2 | 0.1 |
| Germany | 3426 | 67.6 | 15.1 | 0.2 | 17.1 | 2839 | 81.6 | 18.2 | 0.2 |
| Ghana | 30 | 43.3 | 26.7 | 0.0 | 30.0 | 21 | 61.9 | 38.1 | 0.0 |
| Greece | 375 | 79.2 | 8.5 | 0.0 | 12.3 | 329 | 90.3 | 9.7 | 0.0 |
| Hong Kong | 380 | 71.3 | 15.5 | 0.5 | 12.6 | 332 | 81.6 | 17.8 | 0.6 |
| Hungary | 216 | 76.4 | 6.5 | 0.5 | 16.7 | 180 | 91.7 | 7.8 | 0.6 |
| India | 15190 | 69.5 | 13.3 | 0.0 | 17.2 | 12584 | 83.9 | 16.1 | 0.0 |
| Indonesia | 473 | 61.3 | 17.8 | 0.0 | 20.9 | 374 | 77.5 | 22.5 | 0.0 |
| Iran (Islamic Republic of) | 5332 | 66.7 | 14.7 | 0.0 | 18.6 | 4342 | 81.9 | 18.0 | 0.0 |
| Iraq | 597 | 65.5 | 19.4 | 0.0 | 15.1 | 507 | 77.1 | 22.9 | 0.0 |
| Ireland | 214 | 67.3 | 11.7 | 0.0 | 21.0 | 169 | 85.2 | 14.8 | 0.0 |
| Israel | 401 | 72.8 | 10.0 | 0.0 | 17.2 | 332 | 88.0 | 12.0 | 0.0 |
| Italy | 1950 | 66.9 | 19.1 | 0.2 | 13.9 | 1679 | 77.7 | 22.2 | 0.2 |
| Japan | 2870 | 82.1 | 5.4 | 0.0 | 12.4 | 2513 | 93.8 | 6.2 | 0.0 |
| Jordan | 195 | 80.5 | 8.2 | 0.0 | 11.3 | 173 | 90.8 | 9.2 | 0.0 |
| Kazakhstan | 108 | 77.8 | 10.2 | 0.0 | 12.0 | 95 | 88.4 | 11.6 | 0.0 |
| Kenya | 46 | 78.3 | 13.0 | 0.0 | 8.7 | 42 | 85.7 | 14.3 | 0.0 |
| Korea (Democratic People's Republic of) | 43 | 95.3 | 0.0 | 0.0 | 4.7 | 41 | 100.0 | 0.0 | 0.0 |
| Korea (Republic of) | 2543 | 77.1 | 6.6 | 0.0 | 16.3 | 2128 | 92.1 | 7.8 | 0.0 |
| Kuwait | 36 | 80.6 | 5.6 | 0.0 | 13.9 | 31 | 93.5 | 6.5 | 0.0 |
| Lebanon | 91 | 59.3 | 19.8 | 0.0 | 20.9 | 72 | 75.0 | 25.0 | 0.0 |
| Libya | 41 | 97.6 | 2.4 | 0.0 | 0.0 | 41 | 97.6 | 2.4 | 0.0 |
| Lithuania | 74 | 64.9 | 16.2 | 0.0 | 18.9 | 60 | 80.0 | 20.0 | 0.0 |
| Nacao | 32 | 59.4 | 9.4 | 9.4 | 21.9 | 25 | 76.0 | 12.0 | 12.0 |
| Malaysia | 744 | 58.9 | 23.4 | 0.0 | 17.7 | 612 | 71.6 | 28.4 | 0.0 |
| Mexico | 1116 | 72.3 | 12.2 | 0.1 | 15.4 | 944 | 85.5 | 14.4 | 0.1 |
| Morocco | 429 | 68.3 | 19.3 | 0.0 | 12.4 | 376 | 77.9 | 22.1 | 0.0 |
| Nepal | 67 | 89.6 | 1.5 | 0.0 | 9.0 | 61 | 98.4 | 1.6 | 0.0 |
| Netherlands | 748 | 59.2 | 22.5 | 0.3 | 18.0 | 613 | 72.3 | 27.4 | 0.3 |
| New Zealand | 174 | 73.0 | 16.1 | 0.0 | 10.9 | 155 | 81.9 | 18.1 | 0.0 |
| Nigeria | 398 | 77.1 | 6.3 | 0.0 | 16.6 | 332 | 92.5 | 7.5 | 0.0 |
| Norway | 254 | 66.5 | 19.7 | 0.0 | 13.8 | 219 | 77.2 | 22.8 | 0.0 |
| Oman | 42 | 83.3 | 7.1 | 0.0 | 9.5 | 38 | 92.1 | 7.9 | 0.0 |
| Pakistan | 2980 | 77.1 | 11.3 | 0.0 | 11.6 | 2635 | 87.2 | 12.8 | 0.0 |
| Palestine (State of) | 45 | 88.9 | 0.0 | 0.0 | 11.1 | 40 | 100.0 | 0.0 | 0.0 |
| Peru | 44 | 59.1 | 22.7 | 0.0 | 18.2 | 36 | 72.2 | 27.8 | 0.0 |
| Philippines | 96 | 68.8 | 14.6 | 0.0 | 16.7 | 80 | 82.5 | 17.5 | 0.0 |
| Poland | 924 | 59.6 | 19.3 | 0.0 | 21.1 | 729 | 75.6 | 24.4 | 0.0 |
| Portugal | 404 | 62.1 | 18.8 | 0.0 | 19.1 | 327 | 76.8 | 23.2 | 0.0 |
| Qatar | 31 | 83.9 | 6.5 | 0.0 | 9.7 | 28 | 92.9 | 7.1 | 0.0 |
| Romania | 331 | 56.2 | 21.1 | 0.0 | 22.7 | 256 | 72.7 | 27.3 | 0.0 |
| Russian Federation | 2404 | 74.2 | 12.3 | 0.3 | 13.3 | 2085 | 85.5 | 14.1 | 0.3 |
| Saudi Arabia | 1087 | 76.4 | 13.0 | 0.2 | 10.4 | 974 | 85.3 | 14.5 | 0.2 |
| Serbia | 167 | 58.7 | 26.3 | 0.0 | 15.0 | 142 | 69.0 | 31.0 | 0.0 |
| Singapore | 410 | 72.7 | 9.5 | 0.0 | 17.8 | 337 | 88.4 | 11.6 | 0.0 |
| Slovakia | 107 | 63.6 | 22.4 | 0.9 | 13.1 | 93 | 73.1 | 25.8 | 1.1 |
| Slovenia | 91 | 70.3 | 18.7 | 0.0 | 11.0 | 81 | 79.0 | 21.0 | 0.0 |
| South Africa | 436 | 64.9 | 12.2 | 0.2 | 22.7 | 337 | 84.0 | 15.7 | 0.3 |
| Spain | 1367 | 66.9 | 16.2 | 0.1 | 16.8 | 1138 | 80.4 | 19.5 | 0.1 |
| Sri Lanka | 54 | 77.8 | 16.7 | 0.0 | 5.6 | 51 | 82.4 | 17.6 | 0.0 |
| Sweden | 659 | 69.7 | 15.2 | 0.0 | 15.2 | 559 | 82.1 | 17.9 | 0.0 |
| Switzerland | 556 | 66.7 | 13.5 | 0.0 | 19.8 | 446 | 83.2 | 16.8 | 0.0 |
| Taiwan | 1101 | 76.8 | 9.8 | 0.0 | 13.4 | 954 | 88.7 | 11.3 | 0.0 |
| Thailand | 372 | 69.4 | 18.3 | 0.0 | 12.4 | 326 | 79.1 | 20.9 | 0.0 |
| Tunisia | 434 | 52.3 | 28.8 | 0.0 | 18.9 | 352 | 64.5 | 35.5 | 0.0 |
| Turkey | 2324 | 63.6 | 20.3 | 0.0 | 16.1 | 1950 | 75.8 | 24.2 | 0.0 |
| Ukraine | 375 | 71.2 | 12.3 | 0.0 | 16.5 | 313 | 85.3 | 14.7 | 0.0 |
| United Arab Emirates | 180 | 75.0 | 12.2 | 0.0 | 12.8 | 157 | 86.0 | 14.0 | 0.0 |
| United Kingdom | 3345 | 66.1 | 15.7 | 0.2 | 18.1 | 2741 | 80.6 | 19.2 | 0.2 |
| United States of America | 9629 | 66.1 | 15.6 | 0.1 | 18.2 | 7877 | 80.9 | 19.0 | 0.1 |
| Uruguay | 53 | 79.2 | 13.2 | 0.0 | 7.5 | 49 | 85.7 | 14.3 | 0.0 |
| Uzbekistan | 44 | 86.4 | 2.3 | 0.0 | 11.4 | 39 | 97.4 | 2.6 | 0.0 |
| Vietnam | 516 | 78.5 | 11.0 | 0.0 | 10.5 | 462 | 87.7 | 12.3 | 0.0 |

*Note:* Countries without at least 30 submissions with gender identified and submissions without country information were excluded. Gender refers to self-identified gender of the corresponding author. Gender not specified refers to authors who selected “prefer not to say” or did not answer the question on gender. N=118,989.

Supplemental Table S3. Corresponding authorship by gender and journal category

|  | Considering all submissions | | | | | Considering submissions with gender specified | | | |
| --- | --- | --- | --- | --- | --- | --- | --- | --- | --- |
| Category (No. journals) | No. submissions | % Male | % Female | % Non-binary | Not specified | No. submissions | % Male | % Female | % Non-binary |
| Astronomy and astrophysics (2) | 4024 | 76.5 | 8.7 | 0.1 | 14.6 | 3435 | 89.6 | 10.2 | 0.2 |
| Bioscience (15) | 29,158 | 66.0 | 17.5 | 0.0 | 16.4 | 24,369 | 79.0 | 21.0 | 0.0 |
| Environmental science (9) | 19,197 | 67.1 | 17.6 | 0.1 | 15.2 | 16,271 | 79.1 | 20.8 | 0.1 |
| Interdisciplinary (7) | 9423 | 74.7 | 11.8 | 0.1 | 13.4 | 8157 | 86.3 | 13.6 | 0.1 |
| Materials (22) | 59,685 | 64.9 | 14.9 | 0.0 | 20.2 | 47,636 | 81.3 | 18.7 | 0.1 |
| Mathematics (7) | 15,357 | 74.0 | 10.5 | 0.2 | 15.3 | 13,003 | 87.3 | 12.5 | 0.2 |
| Physics (29) | 58,909 | 72.0 | 13.4 | 0.1 | 14.5 | 50,339 | 84.2 | 15.7 | 0.1 |

*Notes:* Gender refers to self-identified gender of the corresponding author. Some journals contributed to more than one category. N=119,592.

Supplemental Table S4. Corresponding authorship by gender and journal

|  |  | Considering all submissions | | | | | Considering submissions with gender specified | | | |
| --- | --- | --- | --- | --- | --- | --- | --- | --- | --- | --- |
| Journal | Category | # submissions | % Male | % Female | % Non-binary | Not specified | # submissions | % Male | % Female | % Non-binary |
| 2D Materials | Materials | 3062 | 69.1 | 14.1 | 0.1 | 16.8 | 2548 | 83.0 | 16.9 | 0.1 |
| Biofabrication | Bioscience, Materials | 1486 | 62.0 | 22.3 | 0.1 | 15.6 | 1254 | 73.4 | 26.5 | 0.1 |
| Bioinspiration & Biomimetics | Bioscience | 1002 | 72.3 | 12.6 | 0.0 | 15.2 | 850 | 85.2 | 14.8 | 0.0 |
| Biomedical Materials | Bioscience, Materials | 1640 | 55.8 | 27.3 | 0.1 | 16.8 | 1365 | 67.0 | 32.8 | 0.1 |
| Biomedical Physics & Engineering Express | Bioscience, Physics | 1155 | 64.3 | 18.6 | 0.0 | 17.1 | 958 | 77.6 | 22.4 | 0.0 |
| Classical and Quantum Gravity | Astronomy and astrophysics, Physics | 2852 | 77.9 | 7.1 | 0.1 | 14.9 | 2427 | 91.5 | 8.3 | 0.2 |
| Electronic Structure | Materials, Mathematics, Physics | 175 | 68.0 | 10.9 | 0.0 | 21.1 | 138 | 86.2 | 13.8 | 0.0 |
| Engineering Research Express | Interdisciplinary | 1392 | 72.7 | 12.7 | 0.0 | 14.6 | 1189 | 85.1 | 14.9 | 0.0 |
| Environmental Research Communications | Environmental Sciences | 513 | 55.6 | 22.2 | 0.0 | 22.2 | 399 | 71.4 | 28.6 | 0.0 |
| Environmental Research Letters | Environmental Sciences | 5396 | 58.7 | 26.9 | 0.1 | 14.3 | 4625 | 68.4 | 31.4 | 0.2 |
| European Journal of Physics | Interdisciplinary, Physics | 2337 | 77.4 | 10.1 | 0.2 | 12.3 | 2049 | 88.3 | 11.5 | 0.2 |
| Flexible and Printed Electronics | Materials, Physics | 261 | 64.4 | 19.2 | 0.0 | 16.5 | 218 | 77.1 | 22.9 | 0.0 |
| Inverse Problems | Environmental Sciences, Mathematics, Physics | 1134 | 68.5 | 15.0 | 0.1 | 16.4 | 948 | 82.0 | 17.9 | 0.1 |
| IOP SciNotes | Environmental Sciences, Interdisciplinary | 324 | 88.6 | 9.3 | 0.3 | 1.9 | 318 | 90.3 | 9.4 | 0.3 |
| Journal of Breath Research | Bioscience | 416 | 51.4 | 29.6 | 0.0 | 19.0 | 337 | 63.5 | 36.5 | 0.0 |
| Journal of Micromechanics and Microengineering | Materials | 1428 | 69.0 | 10.9 | 0.0 | 20.0 | 1142 | 86.3 | 13.7 | 0.0 |
| Journal of Neural Engineering | Bioscience | 1871 | 64.3 | 22.1 | 0.1 | 13.5 | 1618 | 74.4 | 25.6 | 0.1 |
| Journal of Optics | Physics | 2703 | 67.5 | 16.0 | 0.0 | 16.4 | 2259 | 80.8 | 19.2 | 0.0 |
| Journal of Physics A Mathematical and Theoretical | Mathematics, Physics | 4845 | 76.3 | 8.5 | 0.1 | 15.1 | 4115 | 89.8 | 10.0 | 0.2 |
| Journal of Physics Communications | Physics | 1041 | 75.3 | 10.0 | 0.1 | 14.6 | 889 | 88.2 | 11.7 | 0.1 |
| Journal of Physics D Applied Physics | Bioscience, Environmental Sciences, Materials, Physics | 8986 | 69.8 | 14.0 | 0.0 | 16.2 | 7533 | 83.3 | 16.7 | 0.0 |
| Journal of Physics G Nuclear and Particle Physics | Astronomy and astrophysics, Physics | 1172 | 73.1 | 12.7 | 0.2 | 14.0 | 1008 | 85.0 | 14.8 | 0.2 |
| Journal of Physics Complexity | Interdisciplinary, Physics | 236 | 76.3 | 11.9 | 0.0 | 11.9 | 208 | 86.5 | 13.5 | 0.0 |
| Journal of Physics Condensed Matter | Bioscience, Materials, Physics | 5629 | 70.2 | 13.7 | 0.0 | 16.1 | 4724 | 83.6 | 16.3 | 0.0 |
| Journal of Physics Energy | Environmental Sciences, Physics | 360 | 68.9 | 11.7 | 0.0 | 19.4 | 290 | 85.5 | 14.5 | 0.0 |
| Journal of Physics Materials | Materials, Physics | 509 | 61.9 | 24.2 | 0.0 | 13.9 | 438 | 71.9 | 28.1 | 0.0 |
| Journal of Physics Photonics | Physics | 320 | 72.2 | 13.4 | 0.0 | 14.4 | 274 | 84.3 | 15.7 | 0.0 |
| Journal of Radiological Protection | Bioscience | 940 | 61.2 | 21.8 | 0.0 | 17.0 | 780 | 73.7 | 26.3 | 0.0 |
| Machine Learning Science and Technology | Interdisciplinary | 391 | 77.7 | 14.1 | 0.0 | 8.2 | 359.0 | 84.7 | 15.3 | 0.0 |
| Materials for Quantum Technology | Materials, Physics | 46 | 80.4 | 13.0 | 0.0 | 6.5 | 43.0 | 86.0 | 14.0 | 0.0 |
| Materials Research Express | Materials | 14317 | 54.7 | 12.4 | 0.0 | 32.9 | 9609.0 | 81.5 | 18.4 | 0.0 |
| Measurement Science and Technology | Interdisciplinary | 4703 | 72.6 | 12.3 | 0.0 | 15.1 | 3995.0 | 85.5 | 14.5 | 0.0 |
| Methods and Applications in Fluorescence | Bioscience, Materials | 328 | 54.9 | 22.0 | 0.0 | 23.2 | 252.0 | 71.4 | 28.6 | 0.0 |
| Modelling and Simulation in Materials Science and Engineering | Materials, Mathematics | 1945 | 68.8 | 11.4 | 0.1 | 19.7 | 1561.0 | 85.7 | 14.2 | 0.1 |
| Multifunctional Materials | Materials | 118 | 73.7 | 14.4 | 0.0 | 11.9 | 104.0 | 83.7 | 16.3 | 0.0 |
| Nano Express | Materials | 499 | 79.0 | 18.2 | 0.0 | 2.8 | 485.0 | 81.2 | 18.8 | 0.0 |
| Nano Futures | Materials | 275 | 68.0 | 17.8 | 0.0 | 14.2 | 236.0 | 79.2 | 20.8 | 0.0 |
| Nanotechnology | Materials | 9538 | 63.5 | 18.7 | 0.1 | 17.8 | 7845.0 | 77.2 | 22.7 | 0.1 |
| Neuromorphic Computing and Engineering | Bioscience, Interdisciplinary, Materials | 40 | 77.5 | 20.0 | 0.0 | 2.5 | 39.0 | 79.5 | 20.5 | 0.0 |
| New Journal of Physics | Mathematics, Physics | 4244 | 74.1 | 10.1 | 0.2 | 15.6 | 3582.0 | 87.7 | 12.0 | 0.3 |
| Nonlinearity | Environmental Sciences, Mathematics | 2169 | 74.6 | 12.8 | 0.2 | 12.5 | 1898.0 | 85.2 | 14.6 | 0.2 |
| Physica Scripta | Physics | 7312 | 77.5 | 13.3 | 0.0 | 9.1 | 6645.0 | 85.3 | 14.7 | 0.0 |
| Physical Biology | Bioscience, Physics | 443 | 57.3 | 19.9 | 0.0 | 22.8 | 342.0 | 74.3 | 25.7 | 0.0 |
| Physics Education | Physics | 1127 | 77.3 | 12.2 | 0.0 | 10.6 | 1008.0 | 86.4 | 13.6 | 0.0 |
| Physics in Medicine & Biology | Bioscience, Physics | 3809 | 64.8 | 19.3 | 0.1 | 15.8 | 3209.0 | 76.9 | 23.0 | 0.1 |
| Physiological Measurement | Bioscience, Physics | 1351 | 56.5 | 21.5 | 0.0 | 22.0 | 1054.0 | 72.4 | 27.6 | 0.0 |
| Plasma Physics and Controlled Fusion | Physics | 1233 | 75.9 | 11.7 | 0.1 | 12.3 | 1081.0 | 86.6 | 13.3 | 0.1 |
| Plasma Research Express | Physics | 256 | 70.3 | 14.5 | 0.0 | 15.2 | 217.0 | 82.9 | 17.1 | 0.0 |
| Plasma Sources Science and Technology | Physics | 1638 | 77.6 | 12.1 | 0.1 | 10.1 | 1472.0 | 86.3 | 13.5 | 0.1 |
| Progress in Biomedical Engineering | Bioscience | 62 | 58.1 | 29.0 | 0.0 | 12.9 | 54.0 | 66.7 | 33.3 | 0.0 |
| Progress in Energy | Environmental Sciences | 57 | 64.9 | 19.3 | 0.0 | 15.8 | 48.0 | 77.1 | 22.9 | 0.0 |
| Quantum Science and Technology | Mathematics, Physics | 845 | 78.9 | 10.7 | 0.5 | 9.9 | 761.0 | 87.6 | 11.8 | 0.5 |
| Reports on Progress in Physics | Environmental Sciences, Physics | 258 | 73.6 | 8.5 | 0.0 | 17.8 | 212.0 | 89.6 | 10.4 | 0.0 |
| Semiconductor Science and Technology | Materials, Physics | 2632 | 66.9 | 18.0 | 0.0 | 15.0 | 2237.0 | 78.7 | 21.2 | 0.0 |
| Smart Materials and Structures | Materials | 4554 | 73.2 | 12.7 | 0.0 | 14.1 | 3911.0 | 85.2 | 14.8 | 0.0 |
| Superconductor Science and Technology | Materials | 1349 | 76.7 | 11.0 | 0.2 | 12.1 | 1186.0 | 87.3 | 12.5 | 0.3 |
| Surface Topography Metrology and Properties | Materials | 868 | 78.9 | 9.6 | 0.0 | 11.5 | 768.0 | 89.2 | 10.8 | 0.0 |

# *Notes:* Gender refers to self-identified gender of the corresponding author*.* N=119,592.

Figure S1. Boxplot of percent of submissions with male corresponding author, by journal category.

*Notes*: Boxplots consist of values for each journal’s percent of male authorship, of manuscripts with gender identified. Some journals are in more than one category. The box reflects the interquartile range (25^th^ to 75^th^ percentile); the horizontal line reflects the median; the “X” reflects the mean; vertical lines extend to 1.5 times the interquartile range outside the 25^th^ and 75^th^ percentiles; dots reflect values outside the range shown by vertical lines. (N=163,210 as some papers contributed to more than one discipline)


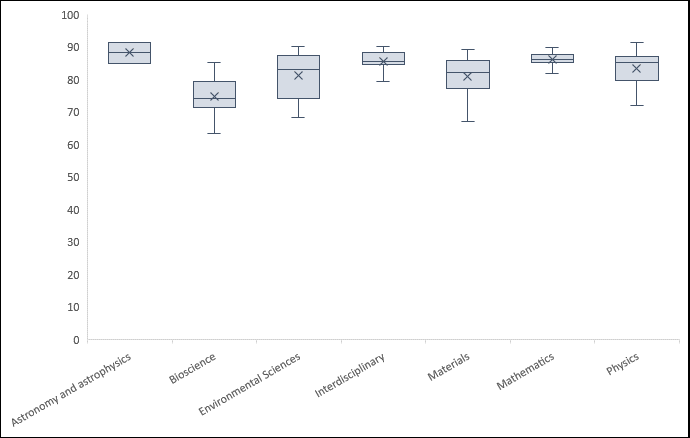


Interdisciplinary

Materials

Mathematics

Physics

Astronomy and Astrophysics

Biosciences

Environmental Science
